# Assessment of Toxic Metals (Al, Cd, Pb) and Trace Elements (B, Ba, Co, Cr, Cu, Fe, Mn, Mo, Li, Zn, Ni, Sr, V) in the Common Kestrel (*Falco tinnunculus*) from the Canary Islands (Spain)

**DOI:** 10.1007/s12011-021-02974-x

**Published:** 2021-10-21

**Authors:** María Rodríguez-Álvarez, Soraya Paz, Arturo Hardisson, Dailos González-Weller, Carmen Rubio, Ángel J. Gutiérrez

**Affiliations:** 1 Toxicology Area, University of La Laguna, La Laguna, 38071 Santa Cruz de Tenerife, Spain; 2 Health Inspection and Laboratory Service, Canary Health Service, 38006 Santa Cruz de Tenerife, Canary Islands Spain

**Keywords:** Canary Islands, *Falco tinnunculus*, ICP-OES, Toxic metals, Trace elements, Biomonitoring

## Abstract

The monitoring of trace elements and toxic metals in apical predators of the trophic chain provides data on the degree of contamination in ecosystems. The common kestrel is one of the most interesting raptors in this respect in the Canary Islands; therefore, the study of the levels of trace elements and toxic metals in this species is of much scientific value. The content of trace elements and toxic metals (B, Ba, Co, Cr, Cu, Fe, Mn, Mo, Li, Zn, Ni, Sr, V, Al, Cd, Pb) was determined in the liver, muscle, and feathers of 200 specimens of common kestrel carcasses (*Falco tinnunculus canariensis*) from Tenerife. Cr (0.82 ± 2.62 mg/kg), Cu (11.82 ± 7.77 mg/kg), and Zn (198.47 ± 520.80 mg/kg) are the trace elements that stand out in the feather samples; this may be due to their affinity for the pigments that give them their coloring. Li was noteworthy in the liver samples (8.470 ± 5.702 mg/kg). Pb stood out in the feathers (4.353 ± 20.645 mg/kg) > muscle (0.148 ± 0.095 mg/kg) > liver (0.187 ± 0.133 mg/kg). The presence of metals in feathers correlates with recent exposure and reflects environmental contamination. When using raptor feathers as indicators of metal contamination, it is important to know what the levels of each metal signify. The analysis of the different tissues and organs of raptors, such as the common kestrel, provides valuable information on the degree of environmental contamination of the ecosystem in which it lives. Gender was not an influencing factor in this study.

## Introduction

Metals are natural constituents of all ecosystems and are present in different concentrations in soils, plants, and animals. These originate from natural processes (erosion, sedimentation, and decomposition), or as a consequence of anthropogenic activities (industrialization, combustion, smelting processes, traffic, mining, agricultural runoff, oil activity, etc.), with humans being mainly responsible for the significant increase of metals in the environment [3, 5, 11]. As a result of increased urbanization and industrialization, large amounts of toxic metals are continuously being introduced into ecosystems, negatively affecting their stability, and having an impact at the ecological level [56].

The mobility, bioavailability, and the impact of the toxicity of these metals in the environment depend not only on the total concentration of the toxic but it is also important to include the climatic conditions, the geochemistry of the soil, the chemistry of the water and sediments, the dose, exposure route, form of the metal or metal compound and chemical species, with the ionic form generally being the most toxic [6].

Some metals such as copper (Cu), cobalt (Co), iron (Fe), manganese (Mn), molybdenum (Mo), selenium (Se), and zinc (Zn) are considered important micronutrients because they are required in high quantities. Other metals such as cadmium (Cd), lead (Pb), or aluminum (Al) are not necessary in these processes. On the contrary, these elements have toxic effects on organisms, and are characterized as polluting or non-essential elements as well as for their bioaccumulation and biomagnification in the trophic chain [11, 21, 41].

Although there are metals with crucial biological functions in plants and animals, sometimes their chemical coordination and oxidation reduction properties have given them an additional advantage whereby they can escape control mechanisms such as homeostasis, transport, compartmentalization, and binding to required cellular constituents. These metals bind with protein sites that are not made for them by displacing the original metals from their natural binding sites, causing cell malfunction and ultimately toxicity [31].

As in other organisms, toxic metals have been associated with health problems in birds, such as reduced body weight, growth rate, and reproductive success. Chronic exposure to these metals can produce negative effects in the behavior of birds, affect their immune systems and other physiological systems such as the endocrine or reproductive ones. In addition to their toxicity and bioaccumulation, metals such as arsenic, cadmium, and copper have also been related to genotoxicity in birds as they have detrimental effects on DNA, and germline mutations have also been documented in birds and mammals in industrial areas [20, 56].

Factors such as age, size, feeding habits, and habitat can affect the accumulation of toxic metals in birds. It is also important to understand the relationship between the concentration of essential metals (Zn, Cu) and non-essential or toxic elements (As, Pb, Cd) with the age of the animal, according to factors such as nutrition and stress. Essential metals such as Zn and Cu have physiological functions, since they are used by the bird organism for processes such as egg development and feather formation, while Pb and Cd are non-essential metals and are strongly linked to pollution [56].

Metal biomonitoring allows the identification of the bioavailability of environmental pollutants from the measurement of chemical residues in tissues or fluids of animals from a specific habitat [10, 11]. Specifically, raptors are an ideal tool to study the environmental quality of the ecosystem through biomonitoring because they are located at the top of the trophic chain, have a wide geographical distribution and they are highly sensitive to changes in the environment [16, 56]. Ecological characteristics of insular environments differ from those of continental ones, leading to adaptations in island-dwelling organisms. These adaptations, through behavioral and/or evolutionary mechanisms, make island species particularly vulnerable to environmental changes, especially those related to anthropogenic activities [24, 69]. However, anthropogenic activities may be asymmetric in a multi-scale approach. As a consequence of direct or indirect anthropogenic activities, many species have become extinct on islands, especially on oceanic islands [70].

The Canary archipelago is made up of oceanic islands which form part of the Mediterranean Basin, an important hotspot in biodiversity [47]. The degree of endemism per square metre of these islands’ biota is one of the highest in the world [8, 33]. There are seven diurnal and two nocturnal species of raptors with seven subspecies in this archipelago [55]. Unfortunately, the populations of raptors in Canary Islands are victims of the harmful influence of anthropogenic activities such as destruction of their habitats, malicious or accidental poisoning, the use of pesticides and rodenticides, toxic discharges, atmospheric contamination or collision with vehicles [2, 13, 45],Ruiz Suárez et al [62]).

However, despite the importance of raptors as suitable sentinels of the environmental contamination, there are no studies concerning heavy metal bioaccumulation in Canary raptors. One of three species of Falconidae which inhabits the Canary Islands is the common kestrel *Falco tinnunculus* (hereafter kestrel). It is a small diurnal falcon with sexual dimorphism in plumage and size, not only being the smallest with the adult male also having a grayish head, rump, and tail [66]. In the Canary Islands, the subspecies *F. t. canariensis* inhabits the western and central islands, including Tenerife [36],it is characterized by feeding mainly on insects. However, in terms of biomass, it is an important predator of lizards and, secondarily, of mice [15]. It is one of the most abundant raptors in urban and suburban environments and reproduces in a wide variety of habitats [14].The present study is an analysis of the differences between trace elements and toxic metals in the liver, muscle, and feather tissues of kestrels carcasses (*F. tinnunculus canariensis*) from the island of Tenerife collected between 2003 and 2018.

## Material and Methods

The extraction of muscle and liver tissues was performed with surgical instruments such as scalpels, scissors, and forceps. Subsequently, for its treatment, reagents of analytical quality with a high degree of purity, distilled water, and glass material previously washed with Acationox detergent (Merck, Germany) and distilled water were used. Finally, the samples were stored in polypropylene containers.

### Study Area

The oceanic island of Tenerife is located in the Atlantic Ocean and is part of the Spanish Canary Islands archipelago. It is located 300 km west of the north western African continent and 1300 km south of the Iberian Peninsula. Its geographical position (near the Tropic of Cancer and under the influence of the trade winds), its elevation (highest point: 3,715 m a.s.l.) and the orientation of its mountain systems give rise to a wide variety of meso and microclimates and vegetation [50].

### Samples

**Fig. 1:**
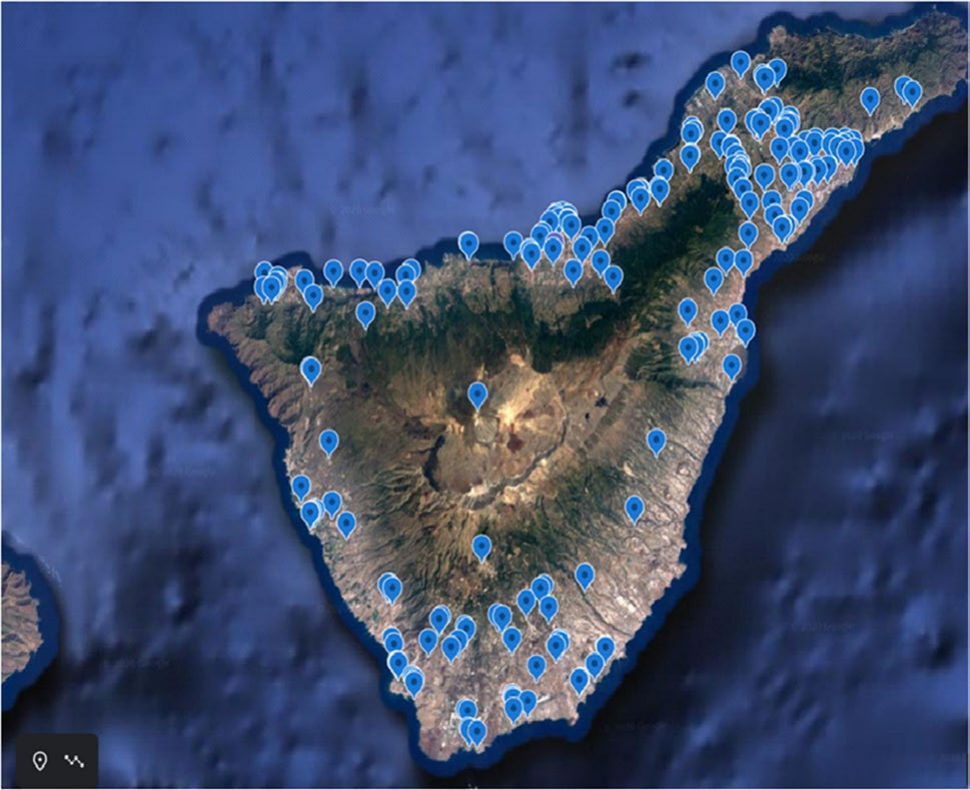
Location of the specimens under study

A total of 200 common kestrel carcasses (*F. tinnunculus canariensis*) were provided by the “La Tahonilla” Wildlife Recovery Center, Tenerife (Canary Islands, Spain). All the individuals came from the island of Tenerife and were collected from 2003 to 2018 and then frozen (Fig. 1). All the individuals analyzed had died mainly from run over, collisions or poisonings (secondary, and direct). The feathers (i.e., 3 rectrices), liver, and muscle (i.e., pectoralis and coracobrachialis) samples were dissected and extracted from each specimen. After dissection, 136 juveniles and 63 adults were determined according to their age, while, when differentiating by sex, 95 were female and 104 were males. Table 1 lists the number of samples used in this study.

**Table 1:** Specimens studied according to their place of origin

Tissue	Location		
	North	South	Metropolitan
Muscle	65	65	58
Liver	64	67	57
Feather	64	67	57

### Treatment of the Samples

Each sample of 0.5 g was placed in porcelain capsules (Staalich, Germany). The feathers were previously washed with double distilled water and cut into small pieces (1–2 cm), as the type of sample is heterogeneous due to the fact they have been exposed to different environmental factors, contamination and dust. Other procedures used were washing which can lead to the leaching of internal concentrations [3, 32, 63]. Once the samples were weighed, they were desiccated in an oven (Nabertherm, Germany) at 70 ± 10 °C for 24 h until their total dehydration [57].

The dehydrated samples were then subjected to incineration in a muffle furnace (Nabertherm, Germany) with a temperature–time program of 450 °C–24 h, with a progressive rise in temperature of 50 °C per hour, until white ash was obtained [49, 60]. At the end of the program, the samples were cooled inside the furnace to avoid contamination [26]. As a result, the organic matter present in the tissue was destroyed.

Specifically, because the liver is a fairly fatty tissue, in order to achieve its total digestion, prior to the incineration stage and after being weighed, a few drops (3–5 mL) of HNO_3_ (65%) were added to the sample and were evaporated on a heating plate (Nabertherm, Germany) at 60 °C [60].

The white ashes obtained from the incineration of the samples were diluted in a 1.5% HNO_3_ solution (Merck, Germany), made up to 25 mL and stored in sterile polypropylene jars. The solution must be transparent and devoid of solid particles [23, 58].

### Analytical Method

The quantification of trace elements and toxic metals was performed using atomic emission spectroscopy, because it is a reference technique for the determination of metals due to its high sensitivity and reproducibility in the results. The type of spectroscopy technique used was inductively coupled plasma optical emission spectrometry (ICP-OES) [60]. The ICP-OES equipment was the ICAP 6300 Duo Thermo Scientific (Waltham, MA, USA) model, with an automatic sampler (model CETAX ASX-520), and the instrumental conditions of the spectrometer were the following: approximate RF power (radio frequency) 1150 W, gas flow (nebulizer gas flow, make-up gas flow) 0.5 L/min, sample injection into the 50 rpm flow pump, 0 s stabilization time. Argon gas (99.999% purity, Air Liquid, Spain) was used for the determination of the elements analyzed by ICP-OES [58].

Table 2 shows the wavelengths (nm) of each element analyzed, as well as the instrumental limits of detection (LOD) and quantification (LOQ), calculated under reproducibility conditions, as three and ten times the resulting standard deviation (SD) from the analysis of 15 targets (IUPAC, [30]).

**Table 2:** Wavelengths, limits of detection and quantification

Element	Wavelength (nm)	Detection limit (mg/L)	Quantification limit (mg/L)
*Toxic metals*			
Al	167.0	0.004	0.012
Cd	226.5	0.0003	0.001
Pb	220.3	0.0003	0.001
*Trace elements*			
B	249.7	0.003	0.012
Ba	455.4	0.001	0.005
Co	228.6	0.0006	0.002
Cr	267.7	0.003	0.008
Cu	327.3	0.004	0.012
Fe	259.9	0.003	0.009
Li	670.8	0.005	0.013
Mn	257.6	0.002	0.008
Mo	202.0	0.0007	0.002
Ni	231.6	0.0007	0.003
Sr	407.7	0.0007	0.003
Zn	206.2	0.002	0.007
V	310.2	0.001	0.005

### Statistical Analysis

The statistical analysis was conducted to determine whether or not there were statistically significant differences (*p* < 0.05) between the three types of samples (liver, feathers, and muscle) of the 200 specimens under study. The Kolmogorov–Smirnov and Shapiro–Wilk tests were applied to determine the distribution of the data. When these did not follow a normal distribution, non-parametric tests such as the Kruskal–Wallis test and the Mann–Whitney *U* test were used. The said statistical tests were performed with the IBM SPSS 22.0 software package.

## Results and Discussion

The toxic metals found at higher concentrations in kestrels carcasses from Tenerife were Al in feathers (298.593 mg/kg dry weight), and Fe in the liver and muscle (181.635 mg/kg dw and 84.921 mg/kg dw, respectively) (Table 3). The highest concentrations were recorded in feathers, except for Li (8.470 ± 5.702 mg/kg dw) in the liver and followed by the muscle (5.827 ± 5.282 mg/kg dw). Specifically, among the toxic metals studied, Al had the maximum value (298.593 ± 139.768 mg/kg dw). The mean content of all the trace elements ranged between 223.714 ± 319.090 mg/kg dw for Fe and 0.260 ± 0.776 mg/kg dw for Co. When comparing the mean metal content obtained between the liver and the muscle, the liver tissue was found to have the highest concentrations of toxic metals and it should also be mentioned that this tissue contained the highest concentrations of most of the trace elements (B, Cu, Mn, Mo, Ni, Fe, Li, Zn). The non-parametric Mann–Whitney *U* test revealed the existence of statistically significant differences in feathers compared to muscle and liver for all metals except for Fe (only in the case of the statistical study between feathers and liver). The mean metal content between the muscle and the liver was also found to differ significantly from one another by *p* < 0.05 except for Ba, Co, Cu, and Sr.

**Table 3:** Mean concentrations and standard deviation (SD) (mg/kg dry weight) in muscle, liver, and feathers

	Tissue	Conc. ± SD (mg/kg dw)	Tissue		Conc. ± SD (mg/kg dw)
Toxic metals					
Al	Liver	27.390 ± 23.475	Pb	Liver	0.187 ± 0.133
	Muscle	18.139 ± 24.096		Muscle	0.148 ± 0.095
	Feather	298.593 ± 139.768		Feather	4.353 ± 20.645
Cd	Liver	0.040 ± 0.062			
	Muscle	0.003 ± 0.015			
	Feather	0.061 ± 0.076			
Trace elements					
B	Liver	0.632 ± 0.511	Mn	Liver	2.698 ± 1.240
	Muscle	0.339 ± 0.401		Muscle	0.760 ± 0.406
	Feather	3.955 ± 4.061		Feather	13.980 ± 17.672
Ba	Liver	1.238 ± 0.875	Mo	Liver	0.352 ± 0.194
	Muscle	1.289 ± 1.466		Muscle	0.013 ± 0.072
	Feather	7.663 ± 6.519		Feather	0.902 ± 6.081
Co	Liver	0.001 ± 0.012	Ni	Liver	0.202 ± 0.278
	Muscle	0.001 ± 0.007		Muscle	0.126 ± 0.293
	Feather	0.260 ± 0.776		Feather	0.781 ± 1.829
Cr	Liver	0.015 ± 0.089	Sr	Liver	2.976 ± 2.955
	Muscle	0.042 ± 0.170		Muscle	3.434 ± 5.659
	Feather	0.823 ± 2.620		Feather	9.573 ± 9.972
Cu	Liver	9.573 ± 7.284	V	Liver	0.279 ± 0.457
	Muscle	8.179 ± 3.613		Muscle	0.370 ± 0.471
	Feather	11.817 ± 7.774		Feather	2.679 ± 10.819
Fe	Liver	181.635 ± 169.217	Zn	Liver	27.602 ± 9.804
	Muscle	84.921 ± 42.298		Muscle	20.463 ± 8.324
	Feather	223.714 ± 319.090		Feather	198.469 ± 520.795
Li	Liver	8.470 ± 5.702			
	Muscle	5.827 ± 5.282			
	Feather	4.411 ± 8.209			

Cd is classified as one of the most dangerous elements, which is persistent in the environment and highly toxic as it affects the endocrine system, kidneys, reproduction, molting, hemoglobin formation and the growth of birds [25]. It can also induce essential element deficiency by competition at active sites of biologically important molecules [37]. Cd concentrations in birds are usually lower in the liver, intermediate in muscle tissue, and high in the kidneys. However, it has been reported that hepatic Cd concentrations can be a good indicator of total exposure to this metal, since up to half of the total cadmium existing in the body can accumulate in this tissue as it is resistant to toxic metal effects [27, 38]. Zaccaroni et al. [71] observed that hepatic Cd concentrations in raptors, specifically in the little owl (*Athene noctua*), of around 0.170 ± 0.008 mg/kg can be considered indicative of chronic exposure and, therefore, have negative effects on animal survival. However, according to the proposed values, the specimens in the present study were found to have a mean hepatic Cd value that can be considered safe (0.040 ± 0.062 mg/kg).

Furthermore, positive correlations between Cd and Zn concentrations have been found for many species of birds. In this respect, a high Zn content reduces the absorption and accumulation of Cd preventing or reducing the toxicity of this metal [39]. It is important to note that the Zn content obtained in the present study was notably higher than that obtained for Cd. The Cd content in feathers can be clearly attributed to the part deposited on them during their growth, as these feathers are connected to the blood and this metal has a special affinity with the thiol groups present in the sulphur of the keratin protein, or as a consequence of the deposition of this metal from atmospheric pollution [43, 44]. These results are similar to those obtained by Zaccaroni et al. [72]*,* where the mean average liver content of the kestrel was less than 1 mg/kg. Hermoso de Mendoza et al. [27] also determined liver concentrations in raptors from Spain, which in many cases did not exceed 1 mg/kg. The above study reported Cd concentrations in kestrels of 1.243 ± 0.749 mg/kg. On the other hand, the mean content obtained in the feathers (0.061 ± 0.076 mg/kg) was similar to the range obtained in other raptors studied in Belgium (0.06–0.33 mg/kg) [18]. Grúz et al. [25] observed a mean average Cd content of 0.20 ± 0.18 mg/kg in kestrel feathers, while in the common buzzard *Buteo buteo* the result was 0.09 ± 0.03 mg/kg.

Pb poisoning can cause serious disorders, such as depression, paralysis, leg or wing weakness, and neuropathological damage to the brain (encephalopathy), which is also manifested as a deterioration of the blood–brain barrier, as well as an enlarged gallbladder [39]. In relation to the Pb present in the feathers, it can have, like Cd, both an endogenous and exogenous origin. This endogenously incorporated metal may reflect the content in the blood since once absorbed, it is distributed to the different internal organs and tissues such as the muscle through the bloodstream. However, its concentration decreases progressively and mainly accumulates in bone tissue and feathers. During initial development, these feathers are irrigated by the blood vessels allowing the mobilization of this toxic metal that has the ability to associate and deposit itself with keratin, remaining immobilized in the feathers; in other words, this could be an indicator of contamination by Pb in a relatively short period of time corresponding to the growth period of these feathers. On the other hand, exogenous Pb may be an indicator of air pollution. This toxic is mainly deposited in the environment as a result of industrial, mining, and transport activities. Therefore, the air is the major receptor of this metal, and as a consequence, it can be deposited on the feathers and becomes an indicator of atmospheric pollution in sedentary species [4, 37, 54].

Pain et al. [53] reported ranges of interpretation of Pb levels in the liver of raptors. According to these authors, hepatic Pb concentrations of less than 2 mg/kg (dry weight) are indicative of environmental exposure without toxicological risk for birds. Other authors have described a liver concentration of 6 mg/kg as the high exposure limit to Pb and 30 mg/kg as a potentially lethal level in raptors [67, 68]. The aforementioned studies suggest that liver concentrations of 2–4 mg/kg (dry weight) in Falconiformes indicate that the animals are exposed to environmental levels higher than normal, although without risk to them [27]. Given the results of the present study, it is possible to consider that based on the mean hepatic value obtained (0.187 ± 0.133 mg/kg), the toxicological risk may be non-existent in the specimens studied and that the mean Pb content obtained in feathers (4.353 ± 20.645 mg/kg) has an exogenous origin since high concentrations were detected that may be indicative of an environment highly contaminated by Pb [46].

Furthermore, according to Burger and Gochfeld [9], Pb begins to be harmful in birds when the mean content in feathers oscillates around 4 mg/kg. This means that had the specimens used in the present study been alive today, they would possibly have suffered from certain harmful effects concerning their health because of bioaccumulation. Dauwe et al. [18] obtained similar results in relation to the Pb content in raptor feathers (2.51–9.9 mg/kg). Furthermore, Pb concentrations of 2.10 ± 1.57 mg/kg have been reported in common kestrel feathers in Hungary [25].

Raptors with scavenging habits mainly feed on mammals and birds, but there are also predator species that feed on reptiles, amphibians, fish, and/or insects. Furthermore, the concentration of metals in their prey contributes significantly to their intake. Eagles and other raptors have often been reported to contain high concentrations of Pb that can reach lethal levels, apparently caused by Pb pellets shot at their prey which they then ingest [27, 44]. This may explain why the concentrations of Pb obtained here are notably lower than those found by other authors whose studies are conducted on raptors that ingest prey containing lead shot. For example, Kitowski et al. [39] reported concentrations of more than 180 mg/kg in liver samples of the white-tailed sea-eagle (*Haliaeetus albicilla*), which is probably a consequence of its feeding habitats in the winter.

It is accepted that the detection of high concentrations of elements as Al, Cd, Cr, Co, Fe, Ni, and Pb in feathers is unlikely to be a consequence of the blood connection during their growth. Thus, these results are thought to be due to direct atmospheric deposition of contaminants or secretions from the uropygial gland on feathers during grooming [18]. Experimental studies carried out with the common starling (*Sturnus vulgaris*, [59] and the zebra diamond (*Taeniopygia guttata*, [17] also refer to the deposition of Pb and Cd on the surface of the feather during grooming. Bearing this in mind, one could consider that the mean average content of Al obtained in the feathers analyzed in the study here (298.593 ± 139.768 mg/kg) is mainly due to external atmospheric pollution since most of the Al present in the environment is a consequence of anthropogenic sources including coal combustion, aluminum production, and metal and mineral smelting [52]. Similar mean values have been recorded in Bonelli’s eagle *Aquila fasciata* feathers from southern Portugal (285.93 ± 450.37 mg/kg) [7]. Adout et al. [1] found a mean Al content in sparrow feathers of 154 ± 19 mg/kg and crow feathers of 153 ± 40 mg/kg from industrial areas. The aforementioned results are markedly lower than those found in the present study indicating that there may be atmospheric pollution caused by Al on the island of Tenerife.

A Swedish study conducted by Ek et al. [19] showed that in the case of the peregrine falcon (*Falco peregrinus*) and Eurasian sparrow hawk (*Accipiter nisus*), Pb contamination is both external and internal, contamination by Zn is internal, and contamination by Cd and Cu is predominantly internal.

Industrial activity in Tenerife is basically made up of small and medium-sized businesses located in urban centers, as well as in industrial estates. The municipalities of La Laguna and Santa Cruz de Tenerife are where most of activity takes place as set out in the Special Territorial Plan for the Management of the Road System of the Metropolitan Area of Tenerife, 2006. There is a wide variety of industrial activity on the island, but these are not particularly important compared to the oil refinery in the capital, Santa Cruz de Tenerife [65].

López-Villarrubia et al. (2008) conducted a study based on the characterization of air pollution in the city of Santa Cruz de Tenerife. The study identified an air pollution pattern characterized by a strong influence of its geographical location (proximity to the largest desert areas of the African continent), terrain, and meteorology. All these factors determine an air quality profile marked by episodes with high levels of particles of natural origin, as well as a pattern of urban-industrial pollution. This may explain why the mean concentrations of Pb and Al were higher in the feathers than in the rest of the tissues analyzed.

Some trace elements are essential and therefore necessary for metabolism, but they can cause adverse effects when their concentrations increase in the body. These elements are required by each tissue to perform various functions, for example, they are necessary for the gradual increase of the movement of the chicks, for the formation and growth of feathers, even for ossification related to bone growth. These metals are not toxic. However, the toxicity of microelements and trace elements has been demonstrated in birds at certain concentrations [37]. The industrial use of fossil fuels and minerals, the use of insecticides and inorganic fertilizers in agriculture, and the accumulation of toxic wastes in urban areas contribute to increasing the levels of these elements above the concentration naturally present in the environment [1].

Zinc (Zn) is an essential element that exists in significantly higher quantities than the rest of the microelements and trace elements studied. Although it is an important component of the enzymes responsible for protein and carbohydrate metabolism [37], this metal mainly accumulates in large quantities in feathers since it is required for their development [5]. The fact that the feathers were found to have higher concentrations of Zn (198.469 ± 520.795 mg/kg) may be because this metal is involved in their pigmentation [29]. Most animals can tolerate a moderate excess of Zn in their diet and regulate the levels in their bodies effectively. Due to this ability, high concentrations of Zn are not alarming from a toxicological point of view, although homeostasis mechanisms can fail when concentrations are extremely high [64]. On the other hand, the mean average hepatic Zn content observed in the present study (27.602 ± 9.804 mg/kg) may be due to the ability of this metal to combine with metallothionein compounds in the liver [44]. As regards the mean Zn content in muscle (20.463 ± 8.324 mg/kg), this was similar to that obtained by Horai et al. [28] in other species of raptors (36.1–78.3 mg/kg).

In the specimens of kestrel studied here, a relatively high mean Fe content was detected in all tissues compared to the other analyzed metals. The high accumulation of Fe in the liver may be indicative of bacterial and helminthological infections (Fe storage disease or hemosiderosis) [35, 42]. Although the infection status of the specimens studied is not known, dissections revealed the presence of intestinal and stomachic parasites in some individuals. However, hemosiderosis occurs when Fe concentrations reach many thousands of mg/kg [35, 42]. In the present study, the mean Fe content in the liver was 181.635 ± 169.217 mg/kg, while Kalisińska et al. [35] reported a mean concentration of 6,149 mg/kg in peregrine falcon liver tissue (*Falco peregrinus*). When comparing concentrations, one should not consider that the presence of parasites in the studied corpses is related to the concentrations of Fe obtained, but that it is possibly a consequence of the storage of Fe that takes place through binding with ferritin proteins present in the liver. A physiological level has been described in Falconiformes that varies from 430 to 2300 mg/kg of Fe in liver [42]. Considering this range, it is possible that the studied specimens were subject to some type of disease caused by the deficiency of this metal. On the other hand, the mean Fe content in feathers obtained in the present study (223.714 ± 319.090 mg/kg) is similar to that obtained by Dauwe et al., [18] in raptors (44–328 mg/kg). It has been suggested that the presence of Fe in the feathers is mainly due to an exogenous origin, with metallurgic and oil refining activities being one of the main absorption factors [42].

As for Cu, this element is involved in the formation of several key enzymes for energy release within the cell and contributes to the function of many antioxidants. This metal is used in industry and agriculture, with these activities being the main reason for its increase in the environment [37]. In the study here, the mean hepatic Cu content was 9.573 ± 7.284 mg/kg. Carpené et al. [12] reported the presence of significant amounts of Cu in the liver. Cu, Cd, and Zn induce liver metallothionein to play a fundamental role in the retention of these metals in some species of birds such as the woodcock (*Scolopax rusticola*). The mean content of this metal has been determined in other nocturnal raptor species such as the African grass-owl *Tyto capensis* (18.0 ± 1.78 mg/kg in liver, 19.0 ± 4.52 mg/kg in muscle, and 2.26 ± 0.28 mg/kg in primary feathers) and the common barn owl *Tyto alba* (26.7 ± 12.6 mg/kg in liver, 16.0 ± 0.906 mg/kg in muscle, and 2.29 ± 0.579 mg /kg in primary feathers) [5].

Mn supports the immune system, regulates blood sugar levels, and is involved in energy production and cell reproduction. One of the main sources of Mn in the environment is the burning of diesel fuel. Teratogenic effects (such as micromyelia, twisted limbs, bleeding, and neck defects), behavioral disturbances, altered growth rates, and reduced hemoglobin formation have been associated with sub-lethal Mn exposure in vertebrates [37]. The mean content of Mn in feathers obtained here (13.980 ± 17.672 mg/kg) is similar to that published by Adout et al. [1], 10.8 ± 16.1 mg/kg in sparrows and crows, and by Dauwe et al. [18] who found a concentration of 11.1–15.3 mg/kg in raptors. The result obtained here for muscle (0.760 ± 0.406 mg/kg) is similar to that of another study (0.883–2.07 mg/kg) [28] and that observed by Kalisińska et al. [34] with the mean muscular manganese content reported being less than 0.55 mg/kg. The latter study also found similarities in the mean liver content of Mn (2.8 mg/kg).

Cr is mainly produced by industrial processes. In appropriate amounts, it plays a fundamental role together with insulin in the regulation of blood sugar [37]. The results obtained in this study were similar to those reported by Zaccaroni et al. [72], in which the mean hepatic Cr content did not exceed 0.4 mg/kg. On the other hand, the mean content in feathers obtained in the present study (0.823 ± 2.620 mg/kg) remained in the same range of the values detected in another study on raptors [18] (0.44–3.56 mg/kg). In the case of the mean Cr content in muscle tissue found here (0.042 ± 0.170 mg/kg), the result was similar to that obtained by Horai et al. [28] in five of the thirteen studied raptors (0.0233–479 mg/kg).

It is known that the pigmentation of feathers can influence the endogenous deposition of certain metals. Eumelanin is the most abundant pigment in feathers which provides the brownish-blackish coloration and has great binding capacity with various metal ions. Therefore, feathers containing more eumelanin may have higher concentrations of these metals [18]. Niecke et al. [51] described Cu, Zn, Mn, and Fe as metals with binding capacity to this pigment. However, Lodenius and Solonen [44] included Hg, Cr, As, and Se. Bearing this in mind, some previously mentioned metals are possibly present in high concentrations in the feathers studied here due to the coloration of the plumage of the kestrel [22].

Ni is a widely distributed trace element in the environment, which is released both naturally and by anthropogenic activities. This element plays an important role in the proper functioning of the liver in animals [37] and this may be the reason why the mean liver content obtained (0.202 ± 0.278 mg/kg) is higher than that found in muscle tissue (0.126 ± 0.293 mg/kg) in the present study. This finding is also reported in a study carried out on other raptor species where Ni concentrations in muscle tissue were lower than in liver tissue. For example, the white-tailed sea-eagle had an Ni concentration of 0.040 mg/kg in muscle while this was 0.087 mg/kg in the liver [48]. On the other hand, the mean content obtained here in feathers (0.781 ± 1.829 mg/kg) was similar to that published by Dauwe et al. [18] in other raptors (0.25–2.70 mg/kg).

B is a trace mineral that is involved in the activity of metabolic enzymes, metabolism of steroid hormones, macronutrients (Ca and Mg), and vitamin D. In addition, it can play a fundamental role in the improvement of arthritis (increases regeneration and bone strength), plasma lipid profiles, brain function, and antioxidant capacity [40]. Therefore, the fact that it was found in higher quantities in the liver of young common buzzards (7.56 ± 18.4 mg/kg) than in adults (4.25 ± 4.78 mg/kg) [40] may be explained by its role in bone development. When comparing these results with those obtained in the present study (0.632 ± 0.511 mg/kg), it could be concluded the individuals studied here had a deficiency of this metal which harmed their health.

As regards Co, the results published by Mihaljev et al. [48], in the three types of samples analyzed, these were similar to the Co concentrations found in the present study which did not exceed 0.1 mg/kg. These low concentrations were also obtained in other studies. This is the case of the paper published by Dauwe et al. [18], who also found mean Co contents in raptor feathers of less than the above value.

Regarding the other metals analyzed (Li, Sr, V, Mo, and Ba), few authors include them in studies of birds, and more specifically, in raptors. The mean liver and muscle content of Li (8.470 ± 5.702 mg/kg and 5.827 ± 5.282 mg/kg, respectively) and Sr (2.976 ± 2.955 mg/kg and 3.434 ± 5.659 mg/kg, respectively) found in the present study are similar to those reported by Horai et al. [28] in their study on thirteen species of birds. Their results concluded that Li is a predominant alkali metal over Sr in all tissues and organs of terrestrial birds, which was only detected in one species of aquatic bird (grey heron *Ardea cinerea*), while Sr is an alkaline earth metal that was quantified in higher concentrations in birds that live in aquatic environments.

Ansara-Ross et al. [5] detected Sr in the liver, muscle, and primary feathers of nocturnal raptors such as *Tyto capensis* (0.250 ± 0.07 mg/kg, 0.143 ± 0.036 mg/kg, and 0.742 ± 0.069 mg/kg, respectively) and *Tyto alba* (0.078 ± 0.036 mg/kg, 0.075 ± 0.005 mg/kg, and 0.675 ± 0.181 mg/kg, respectively). The aforementioned study determined the mean content of V as 0.087 ± 0.032 mg/kg in the liver, 0.256 ± 0.213 mg/kg in the muscle, and 0.268 ± 0.028 mg/kg in primary feathers of *Tyto capensis*, and 0.072 ± 0.047 mg/kg in liver, 0.031 ± 0.003 mg/kg in the muscle, and 0.4 ± 0.112 mg/kg in primary feathers of *Tyto alba* [5]. The mean content of V obtained in feathers in the present study was also higher than that obtained in liver and muscle (0.279 ± 0.457 mg/kg in liver, 0.370 ± 0.471 mg/kg in muscle, and 2.679 ± 10.819 mg/kg in primary feathers). The latter two are also similar to those published in a study from Poland on *Falco peregrinus* liver and muscle samples (0.135 mg/kg in liver and 0.283 mg/kg in muscle) [35]. The mean content of Ba (7.663 ± 6.519 mg/kg) and Mo (0.902 ± 6.081 mg/kg) found in feathers in the present study was similar to the range observed in sparrows (4.84–18.3 mg Ba/kg and 0.17–1.23 mg Mo/kg) [1].

## Conclusions

In conclusion, the analysis of the different tissues and organs of raptors, such as the common kestrel, provides information on the degree of environmental contamination of the ecosystem in which they live. The concentration of trace elements and toxic metals in the liver and muscle can be considered an indication of a chronic exposure. This is because the liver is a detoxification organ in the body, while the muscle is a tissue where these toxic elements and metals are deposited and accumulate. In the case of these two tissues, the metal concentrations in the liver samples were predominant. On the other hand, the presence of metals in feathers mainly correlates with recent exposure as some heavy metals have been cited to redistribute from organ tissue and circulate in the bloodstream which can subsequently accumulate in feathers growing at that time. When using raptor feathers as indicators of metal contamination, it is important to know what the levels of each metal are a sign of.
